# Researcher and patient experiences of co-presenting research to people living with systemic sclerosis at a patient conference: content analysis of interviews

**DOI:** 10.1186/s40900-024-00546-6

**Published:** 2024-01-27

**Authors:** Amanda Wurz, Kelsey Ellis, Julia Nordlund, Marie-Eve Carrier, Vanessa Cook, Amy Gietzen, Claire Adams, Elsa-Lynn Nassar, Danielle B. Rice, Catherine Fortune, Genevieve Guillot, Tracy Mieszczak, Michelle Richard, Maureen Sauve, Brett D. Thombs

**Affiliations:** 1 School of Kinesiology, University of the Fraser Valley, 45190 Caen Ave, Chilliwack, BC V2R 0N3 Canada; 2 Faculty of Kinesiology, University of Calgary, Calgary, AB Canada; 3 Faculty of Medicine, University of Toronto, Toronto, ON Canada; 4 Lady Davis Institute for Medical Research, Jewish General Hospital, Montreal, QC Canada; 5 National Scleroderma Foundation, Tri-State Chapter, New York, USA; 6 Department of Psychiatry, McGill University, Montreal, QC Canada; 7 Department of Psychology, McGill University, Montreal, QC Canada; 8 Mood Disorders Treatment and Research Centre, St. Joseph’s Healthcare Hamilton, Hamilton, ON Canada; 9 Department of Psychiatry and Behavioural Neurosciences, McMaster University, Hamilton, ON Canada; 10 Ottawa Scleroderma Support Group, Ottawa, ON Canada; 11 Sclérodermie Québec, Longueuil, QC Canada; 12 Rochester Scleroderma Support Group, National Scleroderma Foundation, New York, USA; 13 Scleroderma Atlantic, Halifax, NS Canada; 14 Scleroderma Society of Ontario, Hamilton, ON Canada; 15 Department of Epidemiology, Biostatistics, and Occupational Health, McGill University, Montreal, QC Canada; 16 Department of Medicine, McGill University, Montreal, QC Canada; 17 Biomedical Ethics Unit, McGill University, Montreal, QC Canada

**Keywords:** Patient engagement, Co-presentation, Patient-oriented, Dissemination, Experiences, Scleroderma

## Abstract

**Background:**

Patient engagement in research is important to ensure research questions address problems important to patients, that research is designed in a way that can effectively answer those questions, and that findings are applicable, relevant, and credible. Yet, patients are rarely involved in the dissemination stage of research. This study explored one way to engage patients in dissemination, through co-presenting research.

**Methods:**

Semi-structured, one-on-one, audio-recorded interviews were conducted with researchers and patients who co-presented research at one patient conference (the 2022 Canadian National Scleroderma Conference) in Canada. A pragmatic orientation was adopted, and following verbatim transcription, data were analyzed using conventional content analysis.

**Results:**

Of 8 researchers who were paired with 7 patients, 5 researchers (mean age = 28 years, SD = 3.6 years) and 5 patients (mean age = 45 years, SD = 14.2 years) participated. Researcher and patient perspectives about their experiences co-presenting and how to improve the experience were captured across 4 main categories: (1) Reasons for accepting the invitation to co-present; (2) Degree that co-presenting expectations were met; (3) The process of co-presenting; and (4) Lessons learned: recommendations for co-presenting.

**Conclusions:**

Findings from this study suggest that the co-presenting experience was a rewarding and enjoyable way to tailor research dissemination to patients. We identified a patient-centred approach and meaningful and prolonged patient engagement as essential elements underlying co-presenting success.

**Supplementary Information:**

The online version contains supplementary material available at 10.1186/s40900-024-00546-6.

## Abstract (plain language summary)

Involving patients throughout the entire research process is important to ensure research effectively addresses problems important to patients and that findings are applicable, relevant, and credible. Yet, patients are rarely involved in the dissemination of research. We explored one way to engage patients in dissemination, through co-presenting research. We conducted one-on-one interviews with 5 researchers and 5 patients who co-presented research at a patient conference in Canada. Both researchers and patients indicated that the co-presenting experience was rewarding and enjoyable and a useful way to tailor dissemination to patients. We found that a patient-centred approach and meaningful and prolonged patient engagement were essential elements underlying co-presenting success.

**Supplementary Information:**

The online version contains supplementary material available at 10.1186/s40900-024-00546-6.

## Background

Patient engagement refers to active and meaningful collaboration between researchers and patients across all stages of the research process [1]. Effective engagement is essential to ensure that research questions address problems important to patients, that research is designed in a way that can effectively answer those questions, and that findings are applicable, relevant, and credible [2–4]. Governmental and not-for-profit funding agencies [5–8] emphasize or require patient engagement in research. Benefits to patients from engagement, beyond contributing to improving research, may include increased condition-related knowledge and confidence in coping with one’s condition and positive feelings of giving back to the patient community as a valued member of the research team. [9]

Sharing research findings with research participants is mandated internationally by the Declaration of Helsinki [10]. Sharing research findings may also help build trust in research, increase likelihood of research participation, and contribute to supporting patients to be knowledgeable partners in their health care [11]. Results from a 2019 systematic review of 27 studies on patient engagement in cancer research, however, indicated that patients are most often engaged during early stages of research (prioritizing research topics in 10 studies, defining research topics in 7 studies, developing recruitment strategies in 9 studies) but rarely engaged in dissemination (developing dissemination strategies in 2 studies, dissemination of results in 1 study) [4].

Researchers have documented the process of co-creating dissemination tools with patients including lay summaries [12], infographics and website resources [13], and research reports and publications [9]. Results from a 2022 systematic review found 41 studies that described the experience of patients as partners in research, but none reported on patients co-presenting research [14]. To our knowledge, no studies have focused on the co-presentation of research by researchers and patients. We previously described that researchers and patient partners from the Scleroderma Patient-centered Intervention Network (SPIN) co-presented research directly to other patients at the 2022 Canadian National Scleroderma Conference in Niagara Falls, Ontario [15]. The objective of this study was to gather perspectives from researchers and patients who co-presented research to explore experiences of co-presenting, generate evidence to inform further development of this presentation method, and support others to co-present research findings with patients.

## Methods

We conducted a qualitative interview study adopting a pragmatic orientation [16]. This pragmatic orientation offers the flexibility to utilize analytic procedures and processes that “fit” with research aims, while also ensuring internal coherence [17]. Semi-structured interviews were held with researchers and patients who co-presented research at the 2022 Canadian National Scleroderma Conference. This approach, which centers researchers’ and patients’ unique perspectives, is ideally suited to explore co-presenting as a way to engage patients in dissemination. Qualitative methods can afford deeper insights into patients’ lived experiences and perspectives of what worked and what could be changed to improve the process of co-presenting. Similarly, capturing perspectives from researchers—a perspective often overlooked in patient engagement research—could facilitate corroboration with regard to the process of co-presenting. The Consolidated Criteria for Reporting Qualitative Research [18] and Guidance for Reporting Involvement of Patients and the Public-2 (GRIPP2) [19] guided manuscript preparation (see Additional file 1 for GRIPP2 short form checklist). Research Ethics Board approval was obtained from the Research Ethics Committee of the Centre intégré universitaire de santé et de services sociaux du Centre-Ouest-de-l'Île-de-Montréal (#12-123).

### Setting and participants

The Canadian National Scleroderma Conference is an event for individuals with systemic sclerosis (SSc; commonly called scleroderma) that occurs every two years. SSc is a rare, rheumatic disease characterized by abnormal fibrotic processes affecting multiple organ systems including the skin, lungs, gastrointestinal tract, and heart [20, 21]. The conference provides people with SSc opportunities for education on SSc and its management and engagement in mutual support. SPIN is an international organization comprised of individuals living with SSc (SSc patients), researchers, and healthcare providers that researches problems prioritized by patients [22, 23]. Prior to the 2022 Canadian National Scleroderma Conference, SPIN researchers partnered with patients who had been involved in their respective research projects to co-present methods and/or findings. Co-presented research projects involved creating and presenting a poster tailored for presentation to patients. Posters were presented in a large meeting room, and patient conference attendees could move freely among posters and discuss the posters with presenters. There was however a single exception to this; 1 co-presented research project involved creating and presenting an oral presentation, tailored to patients, to conference attendees. Research topics for presentations covered: anxiety and depression symptoms prior to and during COVID-19, a systematic review of effectiveness of non-pharmacological and non-surgical interventions in SSc, progress of SPIN’s self-management program, results from a trial of the SPIN Support Group Leader EDucation (SPIN-SSLED) Program, SSc patient engagement in SPIN, and a scoping review of oral health in SSc.

All researchers and patients who co-presented were contacted and invited to take part in this study. The Director of SPIN (BDT) contacted potential researchers and patients via email and invited them to contact a member of the study team (KE) if they were interested and willing to participate in an interview. Researchers who co-presented research were supervised SPIN research trainees who had or were currently actively conducting a SPIN research project or the SPIN Director. Patients were SPIN participants and project-specific patient advisors who had been engaged in planning, design, interpretation, and dissemination of their respective SPIN research project. At the time of this study, based on a decision by patient Steering Committee members, patient research team members were not compensated, although this policy has since been revised, and SPIN now compensates patient research participants. Researchers and patients were assured that BDT and other SPIN investigators would not be informed of whether they participated in this study, and that the information they provided would be anonymous.

### Data collection

Participants provided informed written consent. Following this, one-on-one, private interviews were scheduled at participants’ convenience during the 2022 Canadian National Scleroderma Conference, after they had presented their research. Interviews took place in a private hotel room that was used by SPIN to coordinate activities, or an empty conference room, and followed a semi-structured interview guide.^1^ The interview guide was designed by the study team to explore researchers’ and patients’ perspectives on the co-presenting experience, understand the co-presenting process (i.e., preparing the abstract, developing presentation materials, preparing presentations, co-presenting), and gather insights regarding aspects that could be modified in the future to enhance the experience (see Additional file 2). Since this was a new area of research, we developed the guide based on the multiple perspectives of team members and did not pre-identify a theoretical framework. Across all interviews, the interviewer (KE) assured researchers and patients that their responses and identity were confidential and explained that she was there to find out more about their experiences and insights to improve the process. To promote rapport and comfort during interviews, KE, a female medical student with nearly 3 years of involvement with SPIN, graduate course work covering qualitative methodologies, study-specific training, and extensive experience conducting one-on-one interviews, introduced herself and her role and answered any questions the participant had about the interview. Following this, she re-confirmed consent verbally and began the interview. All interviews were audio-recorded using a Sony Digital Flash Voice Recorder (ICD-PX312), field notes were taken, and then interviews were anonymized and transcribed verbatim. No repeat interviews were conducted, and researcher and patient participants were not sent a copy of their transcript to review or verify. Rather, all researcher and patient participants had the opportunity to review and comment on the summarized findings (see below).

### Data analysis

Transcribed interviews were analyzed inductively using conventional content analysis [24]. First, two study team members (AW, KE) read each transcript several times to familiarize themselves with the data. Next, they independently coded transcripts, created labels reflecting key ideas, and sorted the codes into higher order categories. Then, AW and KE met to exchange drafts of the coding scheme and compared and challenged one another’s interpretations of the data. Following this, they generated definitions for each category and selected exemplar quotes from the data to illustrate findings from the interviews. Through this iterative process, a table was drafted, including category labels, descriptions, and representative quotes. To enhance readability, repetitive words, excess information, or irrelevant information within quotes were replaced with “[…]” and participant codes were used (R1, R2, R3, etc. for researchers; and P1, P2, P3, etc. for patients). The table was then shared with other study team members (JN, M-EC, VC, AG, CA, E-LN, DBR, BDT), which included researchers and people with SSc. Study team members critically reviewed and commented on the category table. Following this, a final table was drafted and distributed to all researcher and patient participants to review and comment on.

## Results

Eight researchers were initially paired with 7 patients.^2^ Of these, 6^3^ researchers and 7 patients were contacted to participate in this study, and 5 researchers and 5 patients agreed to participate. Participating researchers were on average aged 28 years (SD = 3.6, range = 23–32 years), and the majority self-reported their sex as female (n = 4) and gender as woman (n = 4). They identified as White (n = 3), Hispanic and White (n = 1), and Middle Eastern (n = 1). Mean age of patients was 45 years (SD = 14.2, range = 22–57), and all indicated they were female (n = 5, 100%) and self-identified as White (n = 4) or mixed racial or ethnic background (n = 1). Two of the patient presenters had presented research previously.

Mean times of researcher and patient interviews were 12 min and 15 min, respectively. Researcher and patient perspectives about their experiences co-presenting and what could be improved were captured across 4 main categories: (1) Reasons for accepting the invitation to co-present; (2) Degree that co-presenting expectations were met; (3) The process of co-presenting; and (4) Lessons learned: recommendations for co-presenting. Additional file 3 contains additional exemplary quotes for each category.

### Reasons for accepting the invitation to co-present

For both researchers and patients, the opportunity to learn and experience something new was a draw to participate. Researchers shared that they were excited by the chance to acquire additional patient-engagement skills and described anticipating learning how to make scientific results more accessible, understandable, and valuable to research users when they accepted the invitation. For example, R2 shared:

*It was really good […] for me to learn how to adjust my poster and my abstract. Because there's […] what you think makes sense, and then as soon as you show it to your patient [partner], they're like “that was too big”, “that's a scientific word”. It's really hard when you've had so many years of training to step out of that. And even if you think you have it, there are still words you miss. Still things that won't make sense. So it's a skill to develop.*

Patients expressed an interest in gaining new skills in distilling, interpreting, and presenting scientific information and having the opportunity to make the connection between research and their lived experience. For example, P3 said: *“[Co-presenting] was fascinating and I think it helped me grow as a patient. I can take the knowledge from co-presenting and can also incorporate it into how I engage with the [specific, ongoing SPIN project].”*

Beyond this, patients shared their enthusiasm to take part and co-present stemmed from their passion for SPIN and patient advocacy. Patients in this study had been personally involved with SPIN as participants, patient advisors, and members of the larger SPIN research team for several years. Their prior positive history with SPIN was described as underlying their enthusiasm to co-present. For example, P1 stated: *“My reasons for agreeing were, first and foremost, I love the SPIN organization. […] But also, I was a participant in a patient advisory board for the [specific SPIN project], and I also took part in the [project] after the trial was over. I really got a lot out of it, and I thought it was important to showcase all of the good things that came of it.”*

### Degree that co-presenting expectations were met

This category captures when researchers and patients shared their perceptions of the co-presenting experience as a whole. Within this category, researchers and patients shared that the experience was a positive experience, and one that they would like to be able to have again. For researchers, co-presenting enabled them to see firsthand the impact of their research and work alongside the people for whom the research was done. Thus, this experience served as a good reminder why they were doing the research in the first place. For example, R1 stated: *“Oh yeah, absolutely [I would agree to co-present again]. Just because it felt a little bit more fulfilling. It was just a really interesting experience to have the patients who benefitted from [specific SPIN project] be able to discuss directly with other patients.”*

For patients, co-presenting was seen as fun and exciting, and an opportunity to feel heard. For example, P4 stated *“It [co-presenting] was a great experience in terms of just connecting better with the [SSc] community and the research community.”*

For both researchers and patients, the opportunity to co-present was viewed as a unique opportunity to make meaning out of their research or lived experience. For both, co-presenting afforded the opportunity to build upon one another’s strengths to enhance the relevancy of the presentation. Researchers shared that this experience was rewarding as it challenged their thinking processes, pushed them to better distill information, and required them to prioritize information important to patients. Both researchers and patients appreciated being able to leverage their respective expertise and work together to create a product that was greater than what either could have done on their own. For example, R2 shared:

*[Co-presenting] was helpful because you know researchers and patients have different expertise. So I have the expertise in the methodology and the science and the patient has the expertise in what it’s like to actually be involved in the study. And given, especially, that it was a patient conference, that’s really helpful. You know that’s what patients want to hear.*

P5 echoed the sentiment of being able to leverage respective expertise when they shared:

*My role was to help presenting in a patient friendly vocabulary, patient friendly way the poster. So to review the terms that might be too scientific for the people with scleroderma or just general people so to make sure it’s understandable, easy to understand. So to reduce the gap between the researcher and to evolve to the clinical practice.*

### The process of co-presenting

Researchers and patients described the process they engaged in, which varied widely across co-presenting dyads (i.e., research and patient pairs), when developing their abstract, poster, and presentation. For both researchers and patients, setting deadlines, defining roles, and getting started right away were important elements of preparing to co-present. For most, the process started with a researcher-patient meeting wherein the researcher took the lead and first reviewed the research project and process and then led a discussion on roles and expectations. For example, R5 stated: *“Yeah so there were actually 2 posters and 1 patient, and we were all working together as a team. So, we set up a meeting and then started by addressing what the patient's role was, what our role was, what the whole process […] could look like.”* For these dyads, agenda setting and structure, including goals for each meeting, preparing for meetings in advance, and setting a number of internal deadlines through the process were deemed helpful.

For 1 dyad, at their initial meeting and through the process of developing presentation materials, a natural unfolding was described as occurring (versus agenda setting and structure from the outset). For example, P2 stated: “*You know, when we actually started [working on] our poster […] we never officially delegated anything. We were just kind of like ‘I'm free’. I feel like both of us would just offer and the work got split relatively evenly.”* The lack of agenda setting and structure did not hinder the process or experience of co-presenting; however, these dyads shared that goals, meeting preparation, and internal deadlines would have likely been helpful, and some guidance for agenda setting and developing an equitable plan to share the workload would be desirable.

Researchers and patients also shared that drafts of written documents were researcher-led and iterated upon many times based on patient feedback. For example, R4 shared: *“She [patient] helped to identify areas that would need improvement or areas where it was hard to understand to the lay person. I developed the abstract on my own and I met again with the patient. So, she helped me identify like certain words or sentences [that needed improving or changing].”*

Similarly, P5 stated: *“I revised the abstract mainly and I asked questions because sometimes I didn't understand the way it was formulated, or maybe there were too many words and they were not necessary to have, to add so many words into the abstract or the posters. So [advise so that] it was end-user, more friendly patient words.”*

There was however one notable exception to this wherein a patient led the writing aspect with support and input from the researcher. This was illustrated when P1 shared:

*They really gave me the space to kind of guide me, but also allowed me the creative freedom as a patient to pull what I wanted to showcase. It was really my abstract […]. I think the thing they did [that was most useful] was they didn’t really just take over and do it for me, because it would have been so easy for them to just do that, because they’ve done a million of them you know?*

With regard to visual aspects of the co-presentation process (e.g., poster), patients took the lead. This was because visual and creative aspects of co-presenting were deemed well-suited for engaged patients. Patients appreciated the opportunity to find innovative ways to present results. For example, P3 shared: “*That [poster preparation] was fun! [Researcher] and I brainstormed a little bit and then I went back to the introduction and some of the [project materials], and I took information from that, and I came with up with some sketches of different ways to present the [specific SPIN project] visually.”*

Finally, this category captures patients’ and researchers’ decisions regarding co-presenting at the conference. Decisions about who would cover what material during the poster presentations and oral presentation came easily and in some cases without discussion. Most researchers and patients divided their presentation, regardless of poster or oral, based on expertise. Within the poster presentations, patients spoke about their lived experiences engaging with the research and highlighted key takeaway findings that they felt would be of interest to patients attending their poster. Researchers spoke about the methods and results. For example, R1 stated:

*We decided that she [patient] would present on the [specific SPIN project], because first of all she came up with the brilliant sort of graphic design for how to represent the project, and also, she was very involved in the project. We decided she would present on that, and I would present on the scientific side, which covered the objectives, the methods, and the preliminary results, and then we both presented on key messages to patients.*

As above, there was one notable exception to this wherein the patient took the lead on the poster presentation—sharing their own perspective and the methods and results, and their researcher partner was nearby (at another poster) if they needed help. P1 shared “*So we didn't really decide [who would present what] […] they put their posters [researcher’s other posters] on either side of the one that we did together so that if I needed any help, they [researcher] could help me. But really, I presented it myself which was completely fine.”*

In the case of the oral presentation, a natural unfolding process occurred wherein the workload was shared equally. Overall, across poster and oral presentations, 3 of the dyads shared they had brief conversations, just prior to presenting their poster, to divide speaking roles; however, some did not have an explicit conversation and naturally spoke to areas within their own expertise.

### Lessons learned: recommendations for co-presenting

This category captures when researchers and patients shared the challenges they faced through the co-presentation process and recommendations they would make for others wishing to co-present. These recommendations included:

(i) Establish a patient-centered plan with set roles, expectations, and flexible deadlines. Researchers and patients emphasized the importance of establishing a flexible, patient-centered plan. Within this recommendation, researchers and patients shared that it is important to allocate time to describe the research process and the process of co-presenting, discuss roles and expectations (e.g., with regards to the division of labor), set flexible deadlines that take into account the patients’ competing demands, and ensure ample time during meetings to afford meaningful input and conversation. For example, R5 stated:

*Giving the co-presenters maybe a meeting where we really kind of discussed the purpose, talk about academic presentations, talk about how we’re trying to change this. You know, more clearly defining for them their [patients] roll up front and then giving them some basis on how to fill that role. Because, like I said, they [patient] did mention that they had never done anything like this, and it was totally outside their wheelhouse. So, they weren’t sure what was expected or what they you know where they could contribute.*

Similarly, P4 shared:

*[I would have liked to know] even how to present. I've never done that. So maybe there's more sharing about what would be involved with co-presenting. […] and about what the expectations are, how you would go about co-presenting, etc. Because I was not knowing what I’m supposed to do or how it's supposed to go.*

(ii) Patients appreciated being engaged meaningfully, early, and often. For example, P1 shared: *“They really let me have my say and took my thoughts and ideas into consideration as a top priority and that gave me the encouragement to voice what I liked, what I didn’t like, and really learn the process. So, if I had to do it by myself, I think I could do a good job.”*

Patients underscored the importance of being involved from the outset (whether as participants, patient advisors, research team members, or otherwise) and meaningfully and cautioned that others should be mindful of tokenism and the need to ensure patients are not used as props. For example, P2 shared:

*SPIN does a really great job of integrating the patient into every step of it which I think is what makes it not like that [prop], because if you were to just say you know ‘oh I created all this research like can you just present with me’ kind of thing then it’s like that’s prop [tokenism]. But, if it’s like, no, this person was involved every step of the way, and went through, they had a choice in in what was presented, […] like they really collaborated with the person, that’s better.*

(iii) Researchers emphasized the need to prepare for technology challenges and varied levels of comfortability with online applications. Within this recommendation, phone calls and in-person meetings were preferred and seen as more effective. For example, R3 shared: *“I guess practicing [was the most challenging thing about presenting] since it was all on Zoom. It was a bit harder for us to really understand how it will work. […].”* In cases where meeting in-person was not possible, proactively preparing for technological challenges was seen as important. This could involve setting time at the beginning of the process for researchers to walk through the technology and platforms that will be used or to brainstorm alternative ways of sharing information (e.g., drawing ideas on paper and sending photos, arranging phone calls for patients to talk through their ideas).

## Discussion

Patient engagement in research is important to ensure research questions address problems important to patients, that research is designed in a way that can effectively answer those questions, and that findings are applicable, relevant, and credible [2–4]. Yet, patients are rarely involved in the dissemination stage of research. For instance, a recent systematic review of 41 studies on the experience of patients as research partners found that patients had mostly positive experiences; but, among the 7 studies where patients were involved in research dissemination, none involved co-presenting research [14]. The present study sought to explore one way to engage patients in dissemination, through co-presenting research. Findings from this study suggest that the co-presenting experience was a rewarding and enjoyable way to tailor research dissemination to patients. We identified a patient-centred approach and meaningful and prolonged patient engagement as essential elements underlying co-presenting success.

Researchers and patients in this study shared that they were drawn to the opportunity to co-present for the chance to learn and develop their skills, underscoring the necessity of further supporting researchers’ patient engagement training and educating patients about how to get involved in the research process. Patients also shared that prior experience and a longstanding relationship with SPIN as patient advisors and members of research teams was an important consideration in deciding to be involved. Indeed, SPIN engages patients in study planning, design, interpretation, and dissemination across all of their studies and is intentional about integrating patients as partners. Patients reported that SPIN’s dedicated patient-centered approach and positive track record of including patients enhanced their feelings of comfort and motivation. For those who do not have established patient advisors (e.g., patient advisory boards), a first step may be to identify and include patients early and often in the research process. This can be done by reaching out to past research participants, connecting with local patient and family experience leads within healthcare systems, or connecting with local supportive care networks.

The process of co-presenting research findings is one that SPIN intends to engage in again and that could be easily adapted by others. The present study identified several key lessons learned that will inform ongoing SPIN engagement and support others to co-present research with patients. First, establish a patient-centered, co-presenting plan at the outset with clearly defined researcher and patient roles and expectations. This aligns with published patient engagement recommendations [4]. Second, engage patients meaningfully, early, and often, so as to limit the likelihood of patients experiencing feelings of tokenism and to enhance buy-in [3]. Third, be prepared to navigate technology challenges and varied levels of comfort with online applications by devoting time at the beginning of the process to discuss technology and meeting preferences and to walk through technology and platforms with patients if needed.

Although this study introduced a method of engaging patients that has not previously been described in publications, there are some limitations to consider. First, given the study team’s and the primary interviewer’s role within SPIN, it is possible that researchers and patients were more inclined to provide positive responses and feedback than they would have been with an independent interviewer. We sought to mitigate this by ensuring confidentiality, designing our interview guide to ask balanced and critical questions, and stressing our desire to learn more and improve the process. Nonetheless, it is possible that patients may have emphasized positive experiences and minimized negative ones. Second, participants in this study were predominantly female and White, and their experiences may not reflect what others would report. Third, we did not gather data to understand the patients’ context and previous experience. Gathering information on patients health, disability, level of education and previous professional experience may have provided additional insight to understanding the findings of this study and could be collected in future similar studies. Fourth, the semi-structured interview guide asked questions to explore researchers’ and patients’ perspectives of the co-presenting experience, understand the co-presenting process, and gather insights regarding aspects that could be modified in the future to enhance the experience. As with all qualitative interviews, is possible that the questions asked influenced the kind of experiences people shared and, thus, the building of our categories. Fifth, since presentations were all at a patient conference, it is unknown to what degree findings would be similar for presentations to other audiences, such as co-presentation to researchers or health care providers. Sixth, it is possible that this group of participants may have needed less training than others might need as they were involved in SPIN research projects and not just a single study. Training needs of patient and researcher co-presenters may depend on experience and should be explored in future studies. Finally, we did not get input from patients who attended the conference and the presentations, thus the impact of co-presenting on patient attendees remains unknown. Nevertheless, patient attendees voted for the best poster presentations, and all 3 award recipients were co-presented projects. In the future, this information could be gathered more systematically.

## Conclusions

Notwithstanding these limitations, researchers and patients described an overwhelmingly positive experience co-presenting research at a patient conference. This experience was described as meeting an unmet need and extending already productive relationships. Clarifying roles and expectations, involving patients over long periods of time meaningfully, and ensuring flexibility and training with varied technology may support the co-presenting process, allow for the dissemination of relevant and valuable research findings to patients, and ensure patient engagement throughout the research process.

### Supplementary Information

**Additional file 1**: GRIPP2 Short Form.

**Additional file 2**: Semi-Structured Interview Guide.

**Additional file 3**: Additional Representative Quotes from Researchers and Patients.

## Data Availability

Data from this study are not available in a public archive. De-identified data will be made available as allowable according to institutional research ethics board standards. Requests should be made by emailing the corresponding author.

## Abbreviations

SSc: Systemic sclerosis
SPIN: Scleroderma Patient-centered Intervention Network
SPIN-SSLED: SPIN Support Group Leader EDucation

## Footnotes

1 One researcher could not meet during the conference, and so their interview was scheduled via Zoom within 2 weeks of the conference.

2 One researcher assisted with creating the presentation but did not co-present with their patient partner. In this case, their patient partner co-presented, with assistance from 2 researchers (who were also co-presenting posters with their own patient partners).

3 Two researchers were not contacted to participate because they were the author conducting the interviews (KE) for this study or the Director of SPIN (BDT).
