# Supplementary material for: Researcher and patient experiences of co-presenting research to people living with systemic sclerosis at a patient conference: content analysis of interviews
Source: Res Involv Engagem. 2024 Jan 27;10:13. doi: 10.1186/s40900-024-00546-6 (PMC10822162; doi:10.1186/s40900-024-00546-6)
Supplement: Supplementary file 1 — Additional file 1: GRIPP2 Short Form. [file 40900_2024_546_MOESM1_ESM.docx]

**GRIPP2 Short Form**

| Section and topic | Item | Reported on page no. (in original submission) |
| --- | --- | --- |
| 1: Aim | Report the aim of PPI in the study | 6 |
| 2: Methods | Provide a clear description of the methods used for PPI in the study | 6-9 |
| 3: Study results | Outcomes—Report the results of PPI in the study, including both positive and negative outcomes | 9-17, Additional File 3 |
| 4: Discussion and  conclusions | Outcomes—Comment on the extent to which PPI influenced the study overall. Describe positive and negative effects | 18-19 |
| 5: Reflections/critical  perspective | Comment critically on the study, reflecting on the things that went well and those that did not, so others can learn from this experience PPI patient and public involvement | 18-20 |

PPI patient and public involvement
