# Supplementary material for: Researcher and patient experiences of co-presenting research to people living with systemic sclerosis at a patient conference: content analysis of interviews
Source: Res Involv Engagem. 2024 Jan 27;10:13. doi: 10.1186/s40900-024-00546-6 (PMC10822162; doi:10.1186/s40900-024-00546-6)
Supplement: Supplementary file 2 — Additional file 2: Semi-Structured Interview Guide. [file 40900_2024_546_MOESM2_ESM.docx]

**Semi-Structured Interview Guide**

Hi, thank you for taking the time to be interviewed today. I have some information that I am going to share with you now just so you are clear as to what your participation involves. Feel free to interrupt me at anytime if you need or want me to slow down or repeat anything.

We would like to understand your experience co-presenting by asking you some questions about to process of preparing to co-present, from working on the abstract and poster, to actually co-presenting, and what you found useful or not. All ethics procedures and assurances remain the same for this sub-study as they do for the larger SPIN research program. Your responses and identity will be kept anonymous. As a member of the research team, I will know your identity, but I will assign a code to your name so that no one else from the research team can identify what you will share with me in the interview today.

Your participation is voluntary, and you can withdraw at any time and without any prejudice to you. In the event that you withdraw, or you are withdrawn from the study, all information collected up until that point for the purpose of this study may be used in order to preserve the scientific integrity of the study. There are no right or wrong answers. Your perspective will help us understand more about the nature of co-presenting, and what may be useful for future endeavours like this. Know that you can always skip a question if it makes you feel uncomfortable or if you’d simply rather not answer it.

This interview will be audio recorded and used only for the purposes of data analysis by the research team. The audio recording will be stored in an encrypted file in an encrypted database. Do you have any questions related to this interview? [if yes, interviewer to answer/address; if no, continue to next question.]

1-Can you tell me a little bit about the project that you co-presented on?

PROBES: What was your role (e.g., researcher, patient)? Can you describe your role/involvement with SPIN?

2-What were your reasons for agreeing to co-present ?

PROBE: Can you describe what this experience has been like for you?

3-Based on your experience co-presenting, can you share with me how you wrote the abstract? What about preparing the poster? How did you decide who would present what information at the conference?

PROBES: Why? How were these decisions made? Who, if anyone took a lead? Can you give me an example?

4-What was the most useful/least useful thing that your co-presenter did? What were your biggest challenges co-presenting?

PROBES: Why? Can you give me an example?

5-In reflecting back, what would have made this process easier or more enjoyable for you?

PROBES: Can you describe that further?

6-Looking ahead, would you agree to co-present again?

PROBES: Why/why not?

7-Lastly, can you share with me what your overall impressions of co-presenting were?

8-Do you have anything else that you would like to share before we conclude this interview?
