# Supplementary material for: Researcher and patient experiences of co-presenting research to people living with systemic sclerosis at a patient conference: content analysis of interviews
Source: Res Involv Engagem. 2024 Jan 27;10:13. doi: 10.1186/s40900-024-00546-6 (PMC10822162; doi:10.1186/s40900-024-00546-6)
Supplement: Supplementary file 3 — Additional file 3: Additional Representative Quotes from Researchers and Patients. [file 40900_2024_546_MOESM3_ESM.docx]

**Additional Representative Quotes from Researchers and Patients**

| **Category label** | **Researcher exemplar quotes** | **Patient exemplar quotes** |
| --- | --- | --- |
| **Reasons for accepting the invitation to co-present**  This category captures when patients and researchers shared the reasons for accepting the invitation to co-present. For both parties, the *opportunity to learn something new* was appealing. Patients expressed an interest in gaining new skills distilling, interpreting, and presenting scientific information, and having the opportunity to make the connection between research and their lived experience. Co-presenting was described by researchers as offering them the opportunity to acquire different and specific skills they had yet to gain in their training. Specifically, researchers described anticipation of learning how to make scientific results more accessible, understandable, and valuable to research users. Within this category, patients also shared that they were keen to co-present to *share findings from a project they were personally involved with or based on a prior, positive history with SPIN*. | **The opportunity to learn and experience something new was a draw to participate** | |
|  | *“It was interesting to share that experience [co-presenting] with a patient that was actually involved in the trial. My co-presenter was one of the facilitators for one of the groups that was part of the feasibility study program. It was great to get her perspective on the program, how the study went. For me it was just very interesting, eye opening.”* -R1  *“Overall, [this experience has been] really positive. The patients were very excited to engage with us, to you know, try something new. This was outside a lot of their wheelhouses, so getting to you know share this sort of usually very academic experience with them and finding ways to make it better or more approachable is you know we had some really great conversations. […] I gained a greater appreciation for how technical we really truly are in these posters. And I think also that there are definitely better, or different, maybe better ways of presenting this information.”* -R5 | *“I think [co-presenting] is absolutely fantastic that they want to have patients much more present in the research. One, I think it bridges a gap personally, like on a personal level individual level. That these individuals are able to get a better understanding of what research is, and researchers learn how to use more person friendly and lay person terms and explanations and everything. So it's helpful on both sides. But also on like a bigger level, if this can become more of a thing, I think it really yeah fills a gap that there's you know, there's this huge gap between researchers and the general public and it's like well how do we fill that?”* -P2  *“So, I think, and I think they are both receptive and I know it was pivoting for them as well, because you know you're gone to school, you're trained to, it's all, it's all about the methods and the technical and that's so now we're, you know, asking to pivot and write from a different perspective. So it was a learning curve I think for everyone.”* -P4  *“[I agreed to co-present at the conference] because it's important for me as a patient to be involved in closing the care gap between the research results and bring them into the clinical practice. Or the what have, and then back on uh the result of the research and the impact on the clinical practice or on an outcome for patients as myself. And also to understand as well what is going on outside in the research, in research activities. So to be close to a researcher and to discuss with the with the researcher, help me understand also more what they are doing.”* -P5 |
|  | -- | **A passion for SPIN and patient advocacy** |
|  |  | *“I was very passionate about this [research project] program I've been waiting for over a year for to get off the ground. So I really wanted to share that and help recruit patients so that we can have more people go through the program and get results.”* -P3  *“I think [co-presenting] is valuable again because of the relationship between students and patients. But then as well as participants if they see that there's patients that are co-presenting, there’s perhaps that sensation too that okay it's not just, I'm not, I don't have to just engage with a student either like it's like there's, it's more approachable, the information is more approachable even if that's a visual or not, I don't know. But I like this sense of that it's a joint thing, it's collaborating. So that again participant delegate see that their community is involved in this right, so it gives it more validation.”* -P4 |
| **Degree that co-presenting expectations were met**  This category captures when patients and researchers shared their perceptions of the co-presenting experience as a whole. Within this category, patients and researchers shared that the experience was *rewarding*. For patients, co-presenting was seen as fun and exciting, and an opportunity to feel heard. For researchers, co-presenting enabled them to see firsthand the impact of their research and work together with the people they were doing the research for, which was a good reminder as to why they were doing the research in the first place. For both patients and researchers, the opportunity to co-present was viewed as a unique opportunity to make meaning (out of their lived experience and research, respectively). Researchers shared this experience was rewarding as it challenged their thinking process, pushed them to distill information, and required they prioritize information deemed important to patients. Both patients and researchers appreciated being able to *leverage their respective expertise* and work together to create a product that was greater than what either could have done on their own. | **It was a positive experience, one that would be nice have again** | |
|  | *“I think it [co-presenting] is something that should be done more, after this experience, it seems to be that it just makes sense to try and communicate this research to people who we are trying to provide research for, so overall just very positive impression and thoughts on the whole process. […] I hope we get to do it again.”* -R1  “*Yeah [I would agree to co-present again]. I just think it was a great experience in general. I usually don't like to work with somebody else, but in this case, I thought it was great.*” -R3  *“With the patients, particularly patients that are so invested in the research […] and they want to help you. But definitely like when people have that disease, they want to get involved it affects them. And so just working with someone on that level is good. It's a lot of fun, it's a good experience. So yeah, I would definitely do it again”* -R2 | *“It was really a learning experience. I had never done an abstract proposal or poster before and I think just being able to put the work in, create the poster, and then stand there and present the poster to the delegates at the conference, like it was really just a full circle kind of experience. […] It was a great experience, I learned a lot and yeah I would do it again”* -P1  *“Yeah definitely [I would agree to co-present again]. […] On a deeper level, I know it's [sharing research findings] important.”* -P2  *“It was really interesting, it helped me to understand [more about research]. It was a really, really, good experience.”* -P5 |
|  | **Co-presenting afforded the opportunity to build upon one another’s strengths, enhancing the relevancy of the presentation** | |
|  | *“They're [patients are] coming around the poster and I can talk about OK well this is what the research involves and then [patient co-presenter] naturally said you know in this this is what it's like it's a patient and it just worked really really well to have that shared experience”* -R2  *“But myself and the other researcher we knew, you know, the projects obviously because we had been the ones to run it from start to finish. And so we really kind of lean on them for help with the interpretation, and the discussion of you know why this is significant why it matters what is it that the patients really need to take away from this. […]Yeah like really talking about uh her experience [was the most useful thing my co-presenter did] like it just gave such a such a practical, it just shows like the strong implications of the project. And not just in other academic talking about academic research like she really gave meaning I felt to the poster like just by talking about her own experience in this topic.”* -R4  *“It was a great experience getting different perspectives because I know as a post doc like I come in with a very different perspective than a patient does, than a clinician might, than even a research assistant might and just getting all of those perspectives and that feedback is a really valuable experience for us, and then probably also for the people who are receiving in the information, because they can see how it's been processed by many different people. And we can all provide, maybe saying the same thing but in different ways. It absolutely changed and enhanced what we're doing, and I think it’s just really great because of who the audience was, that we had the people reflected in the posters and presentations.”* -R5 | *“What I was really happy to hear from them, was their willingness and to really make sure that patients understood the information or that they were presenting the information in a way that was gonna be valuable to the patients. And so, and how to even continue to improve that. So I think there was ah, what I liked about it was that, the importance of the information getting disseminated because I'm sure too like sitting and working on things and not really getting any kind of feedback from the people that are supposed to be impacted by it is like well how do we know what we're doing is even of interest or making gonna make an impact. So I think I think from both of them, just that willingness to really want to that, just to present information in a different manner and how important is, was nice.”* -P4 |
| **The process of co-presenting**  This category captures when patients and researchers shared the steps they took preparing for, and co-presenting. Patients and researchers described the *planning* (or lack thereof) they engaged in when developing their abstract, poster, and presentation. For those dyads who planned, the process typically started with a researcher-patient meeting wherein the researcher took the lead and first explained the research project and process and then led a discussion on roles and expectations. These dyads described having goals in place for each meeting, preparing for meetings in advance, and setting a number of internal (mini) deadlines through the process. This structured plan was deemed helpful by those who used it. For those dyads who did not develop or adhere to a plan, a natural unfolding was described as occurring. However, these dyads expressed that a plan would have been helpful and desired more guidance for developing a plan. Within this category, patients and researchers also shared that *numerous researcher-led iterations* of written documents (e.g., abstracts) were required. There was however one notable exception to this wherein a patient led the writing aspect with support and input from the researcher. With regards to visual aspects of the co-presentation process (e.g., poster), *patients took the lead*. Patients appreciated the opportunity to find innovative ways to present results. Finally, this category captures patients’ and researchers’ decisions regarding co-presenting at the conference. Most patients and researchers divided their presentation up based on expertise. Specifically, patients spoke to their lived experience engaging with the research and highlighted key takeaway findings that they felt would be of interest to patients. Researchers spoke to the methods and results. Similar to above, there was one notable exception to this wherein the patient took the lead on the presentation – sharing their own perspective and the methods and results, and their researcher partner was nearby if they needed help. Few dyads shared that they held a conversation amongst themselves to divvy up speaking roles; however, most did not have an explicit conversation and naturally spoke to areas within their own expertise. | **Setting deadlines, defining roles, and getting started right away were important elements of planning for co-presenting** | |
|  | *“Since at the beginning of the project like my patient was not so familiar with the project, we met and we met twice like to basically write the abstract, we met twice. The first time I introduced them to the project, luckily I had presented at a previous academic conference, like this project. So like I showed them the slides and I like just very informally walked them through this slide through the project like in the clearest way I could and it was pretty clear I think and yeah so they quickly understood the project very well it was not very complicated to get across like what the project was and yeah. And then I showed them I had an academic version of this abstract, I showed them the academic version and I asked them like what they thought about it.”* -R4 | *“[The most useful thing that my co-presenter did during this process was] be very patient with me because I've never I mean I've presented papers in the past academically, but coming up with the poster and having to present a new program is something completely foreign to me. So she really walked me through what to expect and was very supportive with my ideas. Just was extremely patient with me.”* -P3 |
|  | **Drafts of written documents were researcher-led, and iterated upon many times based on patient feedback** | |
|  | *“So basically what I did was I tried to rewrite it in a way that was patient friendly, and then I sent that to [name of patient co-presenter] just to send me feedback to see if there was anything that wasn’t easy to understand, if she felt like the language was too academic.”* -R1  *“We sent initial drafts of the abstract that were very much academic at that point and then just did a lot of emailing back and forth between the three of us, revising and trying to refine. And and it was, you know it's a very iterative process because, you know I've been trained to do these posters in one way, and now we were looking to do them very differently. […]”* -R5 | *“And then we obviously condense that all together and had sent it back and forth to [SPIN coordinator] where he we had to like to simplify a lot of our stuff because I feel like it wasn't very like friendly at first. So, we had quite a few revisions where we had made it a bit more lay term I guess. […]And we had basically put in almost all just our information from our abstract and then simplified it a little bit. And then from there again we had like continuous revisions until there was like, we started with so much wording on our poster at first and then we had to like scrap a ton of the wording and [researcher co-presenter] actually did a really good job at it she took out a ton of the wording and made it look really nice.”* -P2  *“I didn't have a lot of involvement in the abstract itself, I helped with a little bit with the wording because we were only conditionally accepted because it was written very technically and they wanted it more patient friendly. So I kind of help with a little bit of the wording on that but that was about it on the abstract.”* -P3 |
|  | -- | **Visual and creative aspects of co-presenting were well-suited for engaged patients** |
|  |  | *“The end product I think which again was very challenging for the students I'm sure, but it really popped in terms of just visually being able to pick up the key components of the paper. Or if it was, you know infographics it's just easier to visually, you know go by and say “okay I can see, what, happy faces” you know or whatever. You can kind of get a sense through that. To be able to relay that information to the participants.”* -P4  *“[It was a really interesting experience because] also to the for the full graphic to have something really more easy to understand and to be more attractive in the poster. So to keep the attention of the people who are looking at the posters.”* -P5 |
|  | **Decisions about who would cover what material during the presentation came easily and in some cases without discussion** | |
|  | *“And she was also able to just share her experiences as a patient being involved. So whilst I was able to explain more of the technical aspects of the research I guess, she was there to explain what it was like to actually be engaged in projects so that was really really helpful. […] So there was never an active conversation of what we're going to do but there was that kind of established from the beginning that I was going to I guess maybe take the lead and the patient was going to just help out if needed, explain more about what it's like being a participant or a patient in the research and it was almost like an unsaid thing that we're each going to bring our own expertise to discuss what's needed.”* -R2  *“So for example in the background we divided it in such a way that, so yeah it was very easy to divide, because like she would take basically at the end like the sections where it would be relevant for her to talk so anything related to clinical practice, anything related like for example the key message for patients like she would do this. In the background I will go over the technical stuff, like what's been done in the literature and things like that, and she would go over what's happening in clinical practice. Same thing for the objectives and for the methods I would go over the technical objectives so that we aim to do a living systematic review and how we did that, and she would go over the website like and how and how it would impact clinical practice and things like that. And same for the results, I took the results and she took the key message for patients.”* -R4 | *“But we did it so we each had an equal amount of slides [for the oral presentation] to present essentially and then yeah I guess the only thing we actually like fully fully delegated was like I asked if she could do the results because I knew she was way more familiar than I was.”* -P2  *“We decided since I had come up with the tree concept and I was most familiar with the actual program itself I did that portion of it. [Research co-presenter] explained the methodology, again what the feasibility trial looked at, and how we're hoping to roll it out later this year.”* -P3 |
| **Lessons learned: recommendations for co-presenting**  This category captures when patients and researchers shared the challenges they faced through the co-presentation process and recommendations they would make for others wishing to co-present. These recommendations included:  (i) Patients and researchers emphasized the importance of establishing a flexible, patient-centered plan. Within this recommendation, it is important to allocate time to describe the research process and the process of co-presenting, discuss roles and expectations (eg, with regards to the division of labor), set flexible deadlines that take into account the patients competing demands, and ensure ample time during meetings to afford meaningful input/conversation. Further, one participant shared importance of volition and choice, thus keeping a patient-centred focus may involve allowing patients to choose the research topic they are interested in participating in.  (ii) Patients expressed the wish to be engaged meaningfully, early, and often. Within this recommendation, it is important to involve patients within research from the outset (whether as patient partners, researchers or otherwise). Indeed, patients expressed concerns regarding tokenism and the need to ensure patients are not used as props.  (iii) Researchers emphasized the need to prepare for technology challenges and varied levels of comfortability with online applications. Within this recommendation, phone calls and in-person meetings were preferred and seen as more effective. In cases where this is not possible, proactively preparing for technological challenges was seen as important. This could involve setting time at the beginning of the process for researchers to walk through the technology and platforms that will be used or to brainstorm alternative ways of sharing information (eg, drawing on paper and sending photos). | **Establish a patient centred plan with set roles, expectations, and flexible deadlines** | |
|  | *“In terms of developing the abstract [patient] was really efficient. So, every time that I worked with her, she’d already read through the abstract, she’d already reviewed the poster. When we started our conversation, [patient] could just straight away [give her] suggestions […] In the future if we had a schedule ahead of time […] that both the researcher and patient knows […], so both people have clear expectations […], and so everyone knows when they need to be available. So that there’s a little bit more structure to it. […] While it might be hard to judge how long you need with each patient, at least if you have that structure and allocated time ahead of time […] Depending on who your patient is as well, like I don’t think it could be a one-size-fits-all but I think there could be a bit more structure to it.”* -R2  *“If I had just some a bit more structure [it would have been easier]. So because since it’s a new process it was some like I wasn’t sure like how to start. […] [A process for co-presenting would look like] the thing is I think it depends on the project and it depends on how easy it is to understand and how also how engaged the patient has been in the project. […] Like I think we really did it in a very structured and nice way and it was very enjoyable but I think that it depends on the project and it depends how much how much the patient wants to be involved and yeah like things like that like. I don’t think there could be like a general process for doing this since it really varies so launch from patient to patient from project to project from you know so many things.”* -R4 | *“No really it was fine [nothing would have made it easier or more enjoyable for me] and it gives me more than enough time to look at the papers to look at, to be prepared for a meeting. So I had things really well in advance so I was able to manage my time.”* -P5 |
|  | -- | **Engage meaningfully, early, and often** |
|  |  | *“I think being engaged more in the whole process like if the whole study and I don’t know how you do that if you can kind of engage patients in the project from the get go […] you’re in it, so you get you, get the methods, you know the methods, and everything that you’re doing, you don’t have to understand everything, but you’re in, so that when it comes time to this, you can, I would feel comfortable, you know, if someone came up and [researcher co-presenter] said “do you want to take this one” or you know I would say “yeah I’ll do this one and you can ask” you know we could do that. But not, like I didn’t feel comfortable enough trying to do that”* -P4 |
|  | **Prepare for technology challenges and varied levels of comfortability with online applications** | -- |
|  | *“Getting to do it in person, the whole time instead of via zoom [would have made it easier/more enjoyable]. There was definitely just technological limitations sometimes like I had a hard time sharing my screen once, so I had to hold up a sketch on a note pad, but I think overall, I think it went quite smoothy.”* -R1  *“I guess being in person would have been a bit easier. I think just being in person we would be able to actually communicate more about it and we would be able to present with the slides on the same place and all of that I just think that would have made it easier.”* -R3 |  |
